# *Drosophila*'s contribution to stem cell research

**DOI:** 10.12688/f1000research.6611.2

**Published:** 2016-08-02

**Authors:** Gyanesh Singh

**Affiliations:** 1 School of Biotechnology and Biosciences, Lovely Professional University, Phagwara, Punjab, India

**Keywords:** Stem cell, Drosophila, Invertebrate stem cell

## Abstract

The discovery of *Drosophila* stem cells with striking similarities to mammalian stem cells has brought new hope for stem cell research. Recent developments in *Drosophila* stem cell research is bringing wider opportunities for contemporary stem cell biologists. In this regard, *Drosophila* germ cells are becoming a popular model of stem cell research. In several cases, genes that controlled *Drosophila* stem cells were later discovered to have functional homologs in mammalian stem cells. Like mammals, *Drosophila* germline stem cells (GSCs) are controlled by both intrinsic as well as external signals. Inside the *Drosophila *testes, germline and somatic stem cells form a cluster of cells (the hub). Hub cells depend on JAK-STAT signaling, and, in absence of this signal, they do not self-renew. In *Drosophila*, significant changes occur within the stem cell niche that contributes to a decline in stem cell number over time. In case of aging *Drosophila*, somatic niche cells show reduced DE-cadherin and unpaired (Upd) proteins. Unpaired proteins are known to directly decrease stem cell number within the niches, and, overexpression of *upd *within niche cells restored GSCs in older males also . Stem cells in the midgut of *Drosophila* are also very promising. Reduced Notch signaling was found to increase the number of midgut progenitor cells. On the other hand, activation of the Notch pathway decreased proliferation of these cells. Further research in this area should lead to the discovery of additional factors that regulate stem and progenitor cells in *Drosophila*.

## Introduction

The fundamental property of stem cells that they can not only differentiate into various types of cells, but can also renew the stem cell population, is the basis of progressive regenerative medicine ^1^. It is normal for some tissues like blood, skin, gut and germ cells to be regularly maintained by stem cell precursors. Stem cell niches control important properties of stem cells including self-renewing potential ^2^. Currently, *Drosophila* germ cells are established as a crucial model of stem cells.

## Analysis of the recent literature

*Drosophila* ovary contains both germline and somatic stem cells that reside within the anterior region of each ovariole ^3^. In an interesting experiment, where individual germaria, free of developing eggs and sheath tissue, were transplanted into the abdominal cavity of a host *Drosophila*, they not only regenerated ovariole-like structures but also maintained oogenesis ^4^. *Drosophila* ovariole usually contains two somatic stem cells (called cystocysts) near the wall of the germarium. Interestingly, somatic stem cells, in this case, not only divide independently of surrounding cells, but also continue to divide in the absence of germline cells ^5^. As asymmetric stem cell division is an important property that enables stem cells to self-renew and differentiate, the balance between symmetry and asymmetry is a tool that enables stem cells to maintain required numbers of progeny cells. An enormous amount of research effort has been directed towards understanding the basis of this asymmetry. Ablation of presumptive germline stem cells (GSCs) near the apical tip blocked the production of new germline cysts, however, previously initiated cysts were able to complete development in this case ^6^. This indicated that development of cysts does not require continued cyst production. More importantly, ablation of a distinct group of somatic cells around GSCs leads to higher egg production ^7^. It has been reported that stem cells adjust their proliferation rate in response to nutrition without changing the number of active stem cells e.g. a protein-rich diet increases the rate of egg production, in this case ^8^.

 Germline and somatic stem cells attach to form a cluster of cells (the hub) in the *Drosophila* testes. The hub expresses a ligand that activates the JAK-STAT signaling cascade ^9^. Without this signal, GSCs do not self-renew, but can differentiate. *Drosophila* bag of marbles ( *bam*) gene is required for the differentiation of daughter cells (cystoblasts) from mother stem cells ^10^. Instead of differentiation, *bam* mutant germ cells proliferated like stem cells. Heat-induced *bam* expression caused elimination of female germinal stem cells while somatic stem cell numbers were not changed ^11^. Interestingly, ectopic *bam* expression had no such consequences on male germline cells indicating *bam*’s potential to regulate oogenesis and spermatogenesis in different ways ^11^. Somatic cyst cells and hub cells express two bone morphogenetic protein (BMP) molecules: Gbb (Glass bottom boat) and Dpp (Decapentaplegic). The Dpp/BMP signal was found to be essential for GSC maintenance ^12^. In absence of BMP signaling, *bam* is upregulated that can cause GSCs to be lost. Mutations in Dpp or its receptor (saxophone) increases stem cell loss and inhibits stem cell division. On the other hand, overexpression of Dpp blocks GSC differentiation ^13^. Interestingly, BMP signaling reduces *bam* expression in ovarian GSCs. Phosphorylated Mad (pMad) is a direct indicator of BMP signaling as C-terminal phosphorylation of Mad by BMP receptor directs Mad toward BMP signaling ^14^. Somatic inner germarium sheath cells failed to divide after removing GSC niches. Hedgehog (Hh) family signaling mediators are known for their important role during *Drosophila* development ^15^. Hedgehog genes were also reported to be crucial for the proliferation of ovarian somatic cells in *Drosophila*. *Drosophila* neuroblasts regulate stem cell growth by separating the growth inhibitor Brat and the transcription factor Prospero into different daughter cells ^15^. Interestingly, mutant Brat or Prospero caused both daughter cells to grow resulting into tumorigenesis ^16^. High levels of Pumilio and Nanos proteins have also been observed in *Drosophila* GSCs ^17^. Lack of zygotic activity of Nanos or Pumilio was found to have a dramatic effect on germline development in female flies. Pumilio mutant *Drosophila* not only failed to maintain stem cells but germline cells also ^17^. Loqs protein was also found to be necessary for embryo survival and GSC sustenance in *Drosophila*. Decrease in stem cell functions could lead to the aging-related decline in tissue maintenance ^18, 19^. Somatic niche cells in testes from aging males show reduced DE-cadherin and unpaired (Upd) proteins ^20^. Inside the *Drosophila* testes, Upd production in hub cells controls stem cell number within the niches, and overexpression of *upd* within niche cells can rescue GSCs even in case of aged males.

The identification of stem cell lineages in the midgut of *Drosophila* is a recent discovery ^21– 23^. A genome-wide transgenic RNAi screen identified 405 genes that regulate intestinal stem cell (ISC) maintenance and differentiation in *Drosophila* intestine ^24^. By integrating these genes into functional networks, it was concluded that factors related to basic stem cell processes are commonly needed in all stem cells, and stem-cell-specific, niche-related signals are required only in the unique stem cell types. Analysis of genetic mosaics revealed that differentiated cells in the midgut epithelium come from a common lineage in *Drosophila* ^25^. Notch signaling controls key events during development. Consistent with its role of regulation of various adult stem cells, diminished notch signaling has been reported to cause increase in the number of precursor cells in the midgut of *Drosophila* ^26^.

## Conclusions

For more than a century, *Drosophila*’s contribution to genetics and developmental biology has been enormous. With its increasing contribution to stem cell research, *Drosophila* consistently proves to be an invaluable model organism. Compared to mammalian model organisms, it is easy and inexpensive to work with *Drosophila*. Furthermore, shorter generation time, small size, and fewer ethical issues makes *Drosophila* an attractive animal model. *Drosophila* germline and midgut stem cells are currently being established as important models of stem cell research. Self-renewal of *Drosophila* GSCs requires both intracellular as well as extracellular signals. Several factors including BMP signals were found to be indispensable for sustaining GSCs in *Drosophila*. Asymmetric division of GSCs to produce and maintain a daughter GSC is regulated by gene expression in adjacent somatic cells also. In *Drosophila*, significant changes occur within the stem cell niche that contributes to a decline in stem cell number over time. These stem cell-related discoveries that were made in *Drosophila*, will surely be helpful for mammalian regenerative medicine, and more work is desperately needed in this area.
