# Supplementary material for: Drosophila's contribution to stem cell research
Source: F1000Res. 2016 Aug 2;4:157. Originally published 2015 Jun 18. [Version 2] doi: 10.12688/f1000research.6611.2 (PMC4490781; doi:10.12688/f1000research.6611.2)
Supplement: Peer review history

# Peer review: Referee response for version 2

**DOI:** 10.5256/f1000research.10085.r15385

**Published:** 2016-08-09

**Authors:** Surajit Sarkar

**Affiliations:** 1 Department of Genetics, University of Delhi, New Delhi, India

I have read this submission. I believe that I have an appropriate level of expertise to confirm that it is of an acceptable scientific standard.

# Peer review: Referee response for version 1

**DOI:** 10.5256/f1000research.7099.r9121

**Published:** 2015-06-30

**Authors:** Surajit Sarkar

**Affiliations:** 1 Department of Genetics, University of Delhi, New Delhi, India

In the manuscript entitled “Drosophila's contribution to stem cell research” by Gyanesh Singh; the author provides an overview of the stem cell research in *Drosophila*. The manuscript provide a brief survey of the recent findings and discuss about various signalling pathways operating in germline stem cell niche. Though it is a good reading and loaded with quality scientific information, some minor corrections may be incorporated as follow:

1. The writing may be improved at some places such as, in second line of abstract “A recent development” should be “Recent developments”.
2. Conclusions of the manuscript may be improved. It should provide a clear and concluding message to the readers.

I have read this submission. I believe that I have an appropriate level of expertise to confirm that it is of an acceptable scientific standard.

# Peer review: Referee response for version 1

**DOI:** 10.5256/f1000research.7099.r9120

**Published:** 2015-06-29

**Authors:** Takashi Adachi-Yamada

**Affiliations:** 1 Department of Life Science, Gakushuin University, Tokyo, Japan

I would like to accept this short review after the author makes improvements described below.

For better contrast to the following sentence, the word “female” should be inserted between the “elimination of ” and “germinal stem cells” in the sentence “Heat-induced bam expression caused elimination of germinal stem cells while somatic stem cell numbers were not changed” in the third section.

The author should reconfirm that the references 12 and 13 are appropriately cited.

The author stated that Phosphorylated Mad (pMad) is an indirect indicator of BMP signaling. However, unlike the case of various reporter genes, I think that pMad is a “direct” indicator because it is directly phosphorylated by BMP type I receptors.

At the position of citation of reference #21, the author should also cite two original papers that first described Drosophila intestinal stem cells, i.e. Ohlstein & Spradling (2006) and Micchelli and Perrimon (2006).

It would be nice if the author concretely indicate some representative factors that are commonly used in all stem cells at the position of reference #22. I think that most of readers in this field have great interest in this recent discovery.

I have read this submission. I believe that I have an appropriate level of expertise to confirm that it is of an acceptable scientific standard.
